# Shotgun metagenomic data of root endophytic microbiome of maize (*Zea mays* L.)

**DOI:** 10.1016/j.dib.2020.105893

**Published:** 2020-06-20

**Authors:** Olubukola Oluranti Babalola, Ayomide Emmanuel Fadiji, Ayansina Segun Ayangbenro

**Affiliations:** Food Security and Safety Niche, Faculty of Natural and Agricultural Sciences, North-West University, Private Mail Bag X2046, Mmabatho, South Africa

**Keywords:** BLAT, Illumina NovaSeq, Maize metagenome, MG-RAST, Plant microbial community, Novel genes

## Abstract

This dataset represents the root endophytic microbial community profile of maize (*Zea mays* L.), one of the largest food crops in South Africa, using a shotgun metagenomic approach. To the best of our understanding, this is the first account showcasing the endophytic microbial diversity in maize plants via the shotgun metagenomics approach. High throughput sequencing of the whole DNA from the community was carried out using NovaSeq 6000 system (Illumina). The data obtained consists of 10,915,268 sequences accounting for 261,906,948 bps with an average length of 153 base pairs and 43% Guanine+Cytosine content. The metagenome data can be accessed at the National Centre for Biotechnology Information SRA registered with the accession number PRJNA607664. Community analysis was done using an online server called MG-RAST, which showed that 0.12% of the sequences were archaeal associated, eukaryotes were 15.06%, while 84.77% were classified as bacteria. A sum of 28 bacterial, 22 eukaryotic and 4 archaeal phyla were identified. The predominant genera were *Bacillus* (16%), *Chitinophaga* (12%), *Flavobacterium* (4%), *Chryseobacterium* (4%), *Paenibacillus* (4%), *Pedobacter* (3%) and *Alphaproteobacteria* (3%). Annotation using Cluster of Orthologous Group (COG) revealed that 41.47% of the sequenced data were for metabolic function, 24.10% for chemical process and signaling, while 17.43% of the sequences were in the poorly characterized group. Annotation using the subsystem method showed that 18% of the sequences were associated with carbohydrates, 9% were for clustering-based subsystems, and 9% contain genes coding for amino acids and derivatives, which might be beneficial in plant growth and health improvement.

**Specification Table**

**Table:** 

Subject	Microbiology
Specific subject data	Environmental Microbiology
Type of data	Raw NGS data
How data were acquired	Shotgun sequencing using NovaSeq 6000 system (Illumina), then structural analysis and annotation of the metagenome through MG-RAST
Data format	Raw data (fastq.gz.file)
Parameters for data collection	Samples from the environment, plant root metagenomes and maize plants.
Description of data collection	Metagenomic DNA extraction from the roots of maize plants from North-West University Farm, Molewane using DNeasy Plant Mini kit (Qiagen), NGS on NovaSeq 6000 system (Illumina) and analysis carried out using MG-RAST
Data location/source	North-West University, Mafikeng, NorthWest, South Africa (S25°47′25.24056″, E25°37′8.17464″).
Data Accessibility	National Centre for Biotechnology Information SRA DIN: PRJNA607664 URL: https://www.ncbi.nlm.nih.gov/sra/PRJNA607664

## Value of the data

- Endophytic microbial communities’ resident in maize plant could serve as a reservoir of plant growth-promoting compounds and novel genes which can help in the growth and health improvement of crops.
- They could serve as an alternative to synthetic fertilizers via the discovery of eco-friendly biofertilizers and potential biocontrol agents in the management of crop diseases.
- Future studies should explore the application and contribution of the novel microbial species and gene discovered in this study for improved agricultural practices.

## Data description

**Fig. 1:**
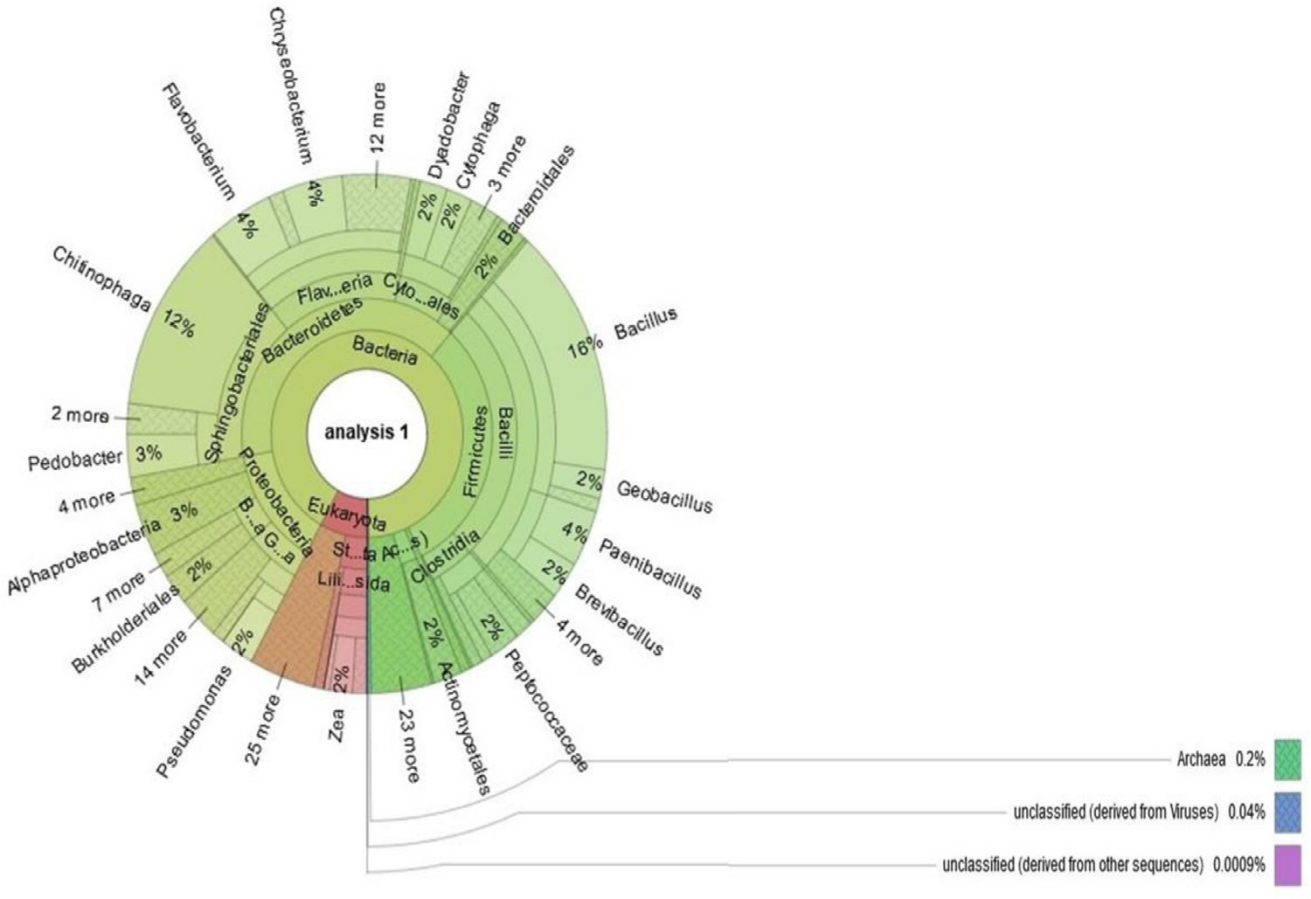
Structure of endophytic microbial communities inhabiting maize plant.

**Fig. 2:**
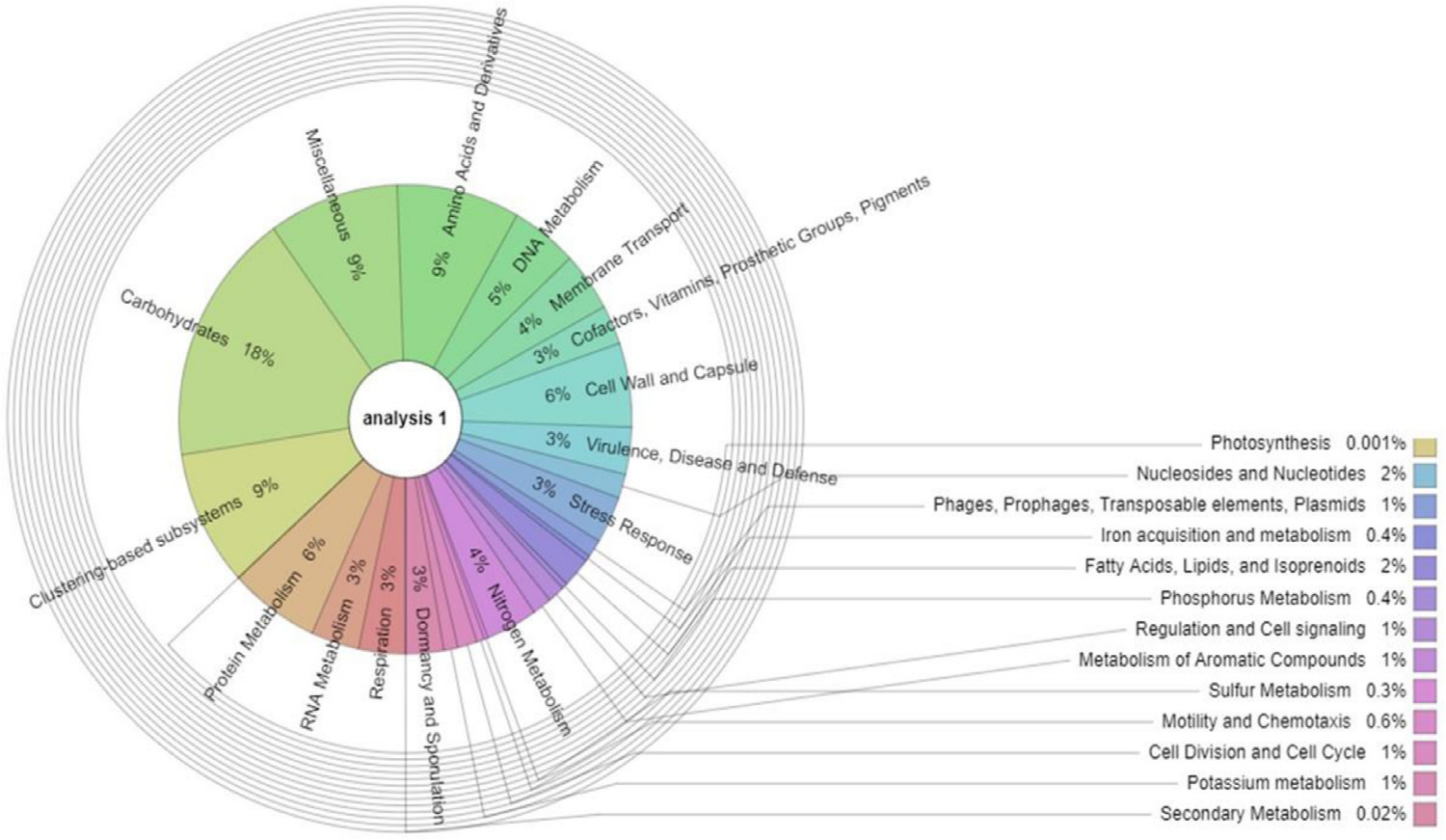
Functional structure of endophytic microbial communities inhabiting maize plants using SEED subsystem.

This dataset contains raw NGS data obtained via shotgun sequencing of maize plant metagenome from South Africa. All datasets obtained in fastq.gz file were deposited at the National Centre for Biotechnology Information SRA database (PRJNA607664). Details of the microbial community and functional structure using SEED subsystem of endophytic microbial communities in maize plants are shown in Fig. 1, Fig. 2 correspondingly.

## Experimental design, materials and methods

Fresh roots of maize plants were collected from the North-West University school farm (S25o47′23", E25o37′15"), Molelwane, Northwest, South Africa. Surface sterilization of the maize roots was carried out using standard methods as described by Correa-Galeote et al. [1], the whole community DNA was extracted from maize plant using Qiagen DNeasy Plant Mini Kit, following guidelines as described by the manufacturer. Shotgun metagenomic sequencing was done using NovaSeq 6000 system (Illumina, USA) following standard methods as provided by the manufacturer. Structural analysis and functional annotation of sequenced data were carried out using an online server called Metagenomics rapid annotation subsystem (MG-RAST) [2] using default specifications. After quality assessment, sequenced data were annotated using a BLAST-like alignment algorithm called BLAT [3], against M5NR database [4] which offers a concise alliance with other numerous databases.

## Declaration of Competing Interest

There is no conflict of interest whatsoever among the authors which could affect the data presented in this paper.

## Appendix Supplementary materials

Image, application 1
